# A rare tumor of the lung: inflammatory myofibroblastic tumor

**DOI:** 10.1186/1746-1596-7-83

**Published:** 2012-07-17

**Authors:** Nawal Hammas, Laila Chbani, Mohammed Rami, Meryem Boubbou, Sara Benmiloud, Youssef Bouabdellah, Siham Tizniti, Mustapha Hida, Afaf Amarti

**Affiliations:** 1 Department of Pathology, HASSAN II University Hospital, Fez, 30000, Morocco; 2 Department of pediatric Surgery, HASSAN II University Hospital, Fez, 30000, Morocco; 3 Department of radiology, HASSAN II University Hospital, Fez, 30000, Morocco; 4 Department of pediatrics, HASSAN II University Hospital, Fez, 30000, Morocco

**Keywords:** Inflammatory myofibroblastic tumor, Lung, Child

## Abstract

Inflammatory myofibroblastic tumor is a rare benign lesion whose tumor origin is now proven. It represents 0.7% of all lung tumors. We report the case of a three-year-old child who suffered from a chronic cough with recurrent respiratory infections. Chest X-ray and computed tomography revealed the presence of a left lower lobe lung mass. After pneumonectomy, histological examination combined with immunohistochemical study discovered an inflammatory myofibroblastic tumor.

The virtual slide(s) for this article can be found here: http://www.diagnosticpathology.diagnomx.eu/vs/8722069326962972.

## Introduction

Inflammatory myofibroblastic tumor (IMT) is a rare benign tumor, accounting for 0.7% of all lung tumors. [1,2] It has been first described by Brunn in 1939. [3] Its origin is unknown, but recent studies have shown that it is a true tumor rather than a reaction process. [1,4]

Its clinical and radiological manifestations are diverse and non specific. That’s why diagnosis is difficult to establish unless a surgical resection is performed. [2,5]

Through this observation, the authors recall the radio-clinical, histopathological, therapeutic aspects, and outcome of this rare tumor.

## Case report

We report the case of a three-year-old child, who has had a chronic cough with recurrent respiratory infections since the age of 1 year.

**Figure 1:**
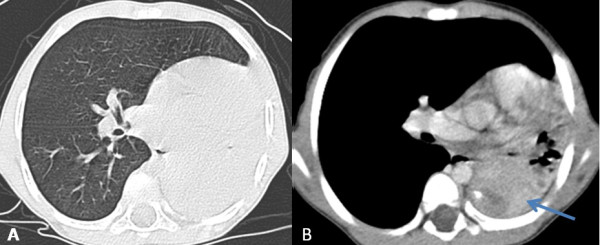
**Chest Computed tomography axial cuts with parenchymal (A) and mediastinal (B) window showing a left lower lobe tumor containing a small calcification, associated with upper lobe atelectasis. **The whole is responsible for the attraction of the mediastinum content to the left side.

**Figure 2:**
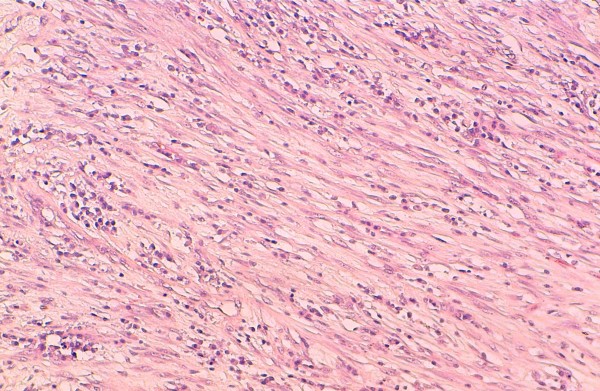
**Spindle cells arrayed in fascicles, mixed with inflammatory cells (medium magnification)**.

**Figure 3:**
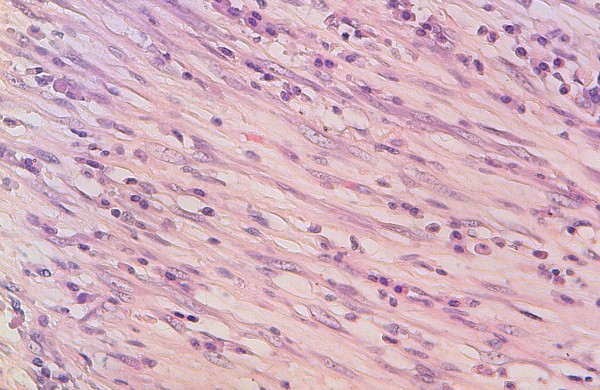
**Proliferation of regular myofibroblasts mixed with lymphocytes and plasma cells (high magnification)**.

**Figure 4:**
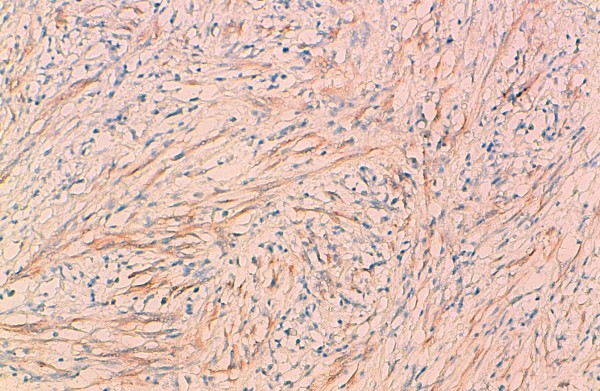
**Immunohistochemical study showing reactivity for ALK1**.

**Figure 5:**
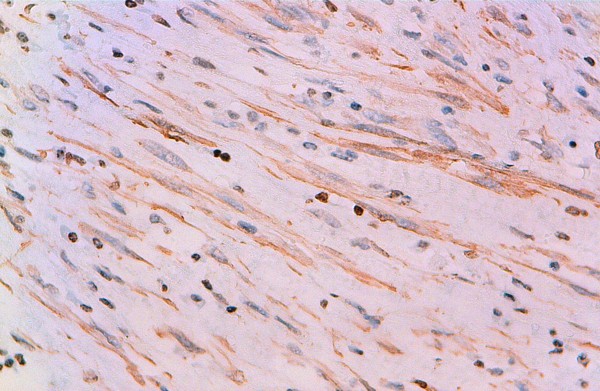
**Immunohistochemical study showing reactivity for AML**.

Chest X-ray showed a homogenous opacity invading the entire left hemithorax. Chest computed tomography (CT) scan showed a left lower lobe tumor with a small calcification, associated with upper lobe atelectasis. This attracted the mediastinum content to the left side (Figure 1). Bronchoscopy showed a complete obstruction of the left main bronchus. The resort to surgery was for diagnostic and therapeutic purposes, and consisted of a left pneumonectomy. On gross examination, the tumor was 8.5 cm in size, firm, whitish and homogeneous. Microscopic examination revealed a proliferation of regular spindle cells arrayed in fascicles, admixed with lymphocytes, plasma cells and eosinophils (Figures 2 and 3). Immunohistochemical analysis showed positive staining for ALK1 (Figure 4), smooth muscle actin (Figure 5), and H-caldesmon. In contrast, the tumor cells were not reactive to PS100. Based on these data, the diagnosis of inflammatory myofibroblastic tumor was retained.

## Discussion

The IMT is a rare benign lesion representing 0.7% of all lung tumors. It was previously called inflammatory pseudotumor, plasma cell granuloma, histiocytoma or fibroxanthoma. [1,2,5,6] It was first described in the lung in 1939 but other extrapulmonary sites were reported [2,4,6]. Völker and al [7] reported a laryngeal IMT and compared it with spindle cell carcinoma. Because of similar morphology of theses lesions, only immunohistochemical investigations allowed the correct final diagnoses. Another case was reported in the urinary bladder by Lekas and al [8]. It was initially misinterpreted as a low-grade leiomyosarcoma of myxoid subtype. Al-Jabri [9] reported another case in the liver in which imaging raised the possibility of metastatic liver disease because of the similarity of appearances between the two pathological entities. Histological examination was necessary for diagnosis. Several other sites were reported including spleen, lymph nodes, esophagus, stomach, salivary glands, breast, epididymis, central nervous system, and soft tissues. [2,4,6] The IMT affects both sexes, at any ages, with a slight predominance in children and young adults. [4,6] In our patient, the tumor was discovered at a very early age.

There are many uncertainties about the pathogenesis of IMT. Several hypotheses have been proposed such as an auto-immune mechanism or infectious origin. Indeed, 30% of cases are closely related to recurrent respiratory infections which are caused by several microorganisms such as Mycoplasma, Nocardia, Actinomycetes, Epstein-Barr and human herpes virus [2,3,5,6,10]. Other studies, however, suggest that it might be a true neoplasm due to the presence, at the myofibroblastic component, of a fusion gene involving the ALK gene, a tyrosine kinase oncogen located on chromosome 2p23, initially found to be arranged in anaplasic large cell lymphomas. This fusion leads to constitutive overexpression of the ALK, causing cell proliferation. [4-6,10,11] In our patient, the tumor is due to overexpression of the ALK, detected by immunohistochemistry, and is probably favored by recurrent respiratory infections.

Patients are afflicted with nonspecific symptoms such as cough, dyspnea, hemoptysis, chest pain, fever and fatigue. Weight loss and anorexia are rare. [2-6] However, most patients are asymptomatic and the tumor is discovered incidentally on a chest X-ray performed for another reason. [1,4,6] The only symptom in our patient was a chronic cough.

Radiological aspects are variable and nonspecific. Most patients (87%) have a mass or a pulmonary nodule, solitary, rarely multiple (5%), measuring 1 to 6 cm in diameter, sharply limited, smooth or bumpy. The lesion is located on the periphery with a predilection for the lower lobes. [2,4,6]

Computed tomography shows the presence of a heterogeneous nodule or mass with variable contrast enhancement. It specifies the tissue or cystic nature of the tumor, its vascular behavior and it assesses the locoregional extension. Calcifications and cavitations are rare. Pleural effusion is seen in less than 10% and atelectasis in 8% of cases. Sometimes the tumor can extend towards the hilum, mediastinum, pleura or diaphragm. [1,2]

The treatment of choice for diagnostic and therapeutic reasons is a complete resection. An incomplete resection increases the risk of recurrence. [1-3,5] Corticosteroids are generally not useful in adults, although good results have been reported in children in cases of unresectable tumors or hilar and mediastinal invasion. Chemotherapy is useful in cases of multifocal, invasive lesions or in cases of local recurrence. [2]

On gross examination, the tumor is well circumscribed but not encapsulated, firm and homogeneous, with sometimes some foci of necrosis. [10]

Because of the diversity of clinical and radiological manifestations of IMT, diagnosis is difficult to establish without chirurgical management. It is therefore based on histological examination of the chirurgical resection specimen that shows a lesion formed of varying proportions of spindle cells of myofibroblastic type, arranged in a fibrous, myxoid or calcified stroma, associated with an inflammatory component predominantly lymphocytic and plasmacytic, with a variable component of eosinophils. [2,4,5,10]

IMT includes three histological subtypes: one is a richly vascularized and myxoïde resembling fasciitis or granulation tissue; onother is a more compact fascicular spindle cell proliferation with variable collagenized regions and lymphoid nodules, resembling fibromatosis, and finally a very sclero-hyaline, slightly cellular pattern, looking more like a desmoid tumor. [4]

Immunohistochemistry showed reactivity for vimentin and smooth muscle actin. Immunohistochemical positivity for ALK is detectable in just over half of the cases with cytoplasmic staining, more rarely at the nuclear membrane. [10,11]

Pathologic differential diagnosis includes organized pneumonia, lymphoma and solitary fibrous tumor. The mode of tumor growth, the low mitotic index, the polyclonality of lymphoid markers and the negativity of CD34 usually remove most of these diagnoses. Other differential diagnoses are desmoid fibromatosis, angiomyofibroblastoma, fibrosarcoma, leiomyoma, and malignant fibrous histiocytoma. [2,4]

Despite IMT is a benign tumor, it is considered by some authors as a low grade tumor because of malignant features such as local invasiveness, recurrence (4% in cases of incomplete resection) or malignant transformation (exceptional). The evolution depends on the tumor size (less than or equal to 3 cm) and the quality of surgical resection. The 5-year survival is 91.3%. [2-5].

## Conclusions

Inflammatory myofibroblastic tumor is a rare benign tumor. Clinical and radiological presentation is variable and nonspecific and the diagnosis is rarely made before chirurgical management. Only histological and immunohistochemical study can confirm the diagnosis. Despite being a benign lesion, its potential for recurrence and local invasion requires complete surgical resection.

## Competing interests

The authors declare that they have no competing interests.

## Authors’ contributions

NH, LC and AA performed the histological examination of the lung and were major contributors to writing the manuscript. SB analyzed and interpreted the patient data. M R is the surgeon who operated on the patient and interpreted the patient data. MB performed the radiological examination. MH, YA, ST assisted in data interpretation and patient follow-ups. All authors read and approved the final manuscript.

## Consent

Written informed consent was obtained from patient's parents for publication of this case report and any accompanying images.
